# hBN Nanoparticle-Assisted Rapid Thermal Cycling for the Detection of *Acanthamoeba*

**DOI:** 10.3390/pathogens9100824

**Published:** 2020-10-07

**Authors:** Abdul Khaliq Rasheed, Ruqaiyyah Siddiqui, Salma Mohammed Kabir Ahmed, Shobana Gabriel, Mohammed Zayan Jalal, Akbar John, Naveed Ahmed Khan

**Affiliations:** 1 Department of New Energy Science and Engineering, School of Energy and Chemical Engineering, Xiamen University Malaysia Campus, Bandar Sunsuria, Sepang 43900, Malaysia; abdulkhaliq.rasheed@xmu.edu.my; 2 Department of Biology, Chemistry and Environmental Sciences, College of Arts and Sciences, American University of Sharjah, Sharjah 26666, UAE; rsiddiqui@aus.edu; 3 Department of Biological Sciences, School of Science and Technology, Sunway University, Bandar Sunway 47500, Malaysia; m.salma2015@hotmail.com (S.M.K.A.); shobygurl.gabriel@gmail.com (S.G.); 4 Department of Mechanical Engineering, Faculty of Engineering, International Islamic University Malaysia, Jalan Gombak 53100, Malaysia; zayan_mohammed@yahoo.co.in; 5 Institute of Oceanography and Maritime Studies, Kulliyyah of Science, International Islamic University Malaysia, Kuantan 25200, Malaysia; akbarjohn@iium.edu.my

**Keywords:** nanoPCR, hexagonal boron nitride, thermal conductivity, *Acanthamoeba*, pathogen, bio-heat transfer

## Abstract

*Acanthamoeba* are widely distributed in the environment and are known to cause blinding keratitis and brain infections with greater than 90% mortality rate. Currently, polymerase chain reaction (PCR) is a highly sensitive and promising technique in *Acanthamoeba* detection. Remarkably, the rate of heating–cooling and convective heat transfer of the PCR tube is limited by low thermal conductivity of the reagents mixture. The addition of nanoparticles to the reaction has been an interesting approach that could augment the thermal conductivity of the mixture and subsequently enhance heat transfer through the PCR tube. Here, we have developed hexagonal boron nitride (hBN) nanoparticle-based PCR assay for the rapid detection of *Acanthamoeba* to amplify DNA from low amoeba cell density. As low as 1 × 10^−4^ wt % was determined as the optimum concentration of hBN nanoparticles, which increased *Acanthamoeba* DNA yield up to ~16%. Further, it was able to reduce PCR temperature that led to a ~2.0-fold increase in *Acanthamoeba* DNA yield at an improved PCR specificity at 46.2 °C low annealing temperature. hBN nanoparticles further reduced standard PCR step time by 10 min and cycles by eight; thus, enhancing *Acanthamoeba* detection rapidly. Enhancement of *Acanthamoeba* PCR DNA yield is possibly due to the high adsorption affinity of hBN nanoparticles to purine (Guanine—G) due to the higher thermal conductivity achieved in the PCR mixture due to the addition of hBN. Although further research is needed to demonstrate these findings in clinical application, we propose that the interfacial layers, Brownian motion, and percolation network contribute to the enhanced thermal conductivity effect.

## 1. Introduction

*Acanthamoeba* is a free-living protozoan widely found in the environment. Despite being ubiquitous, they can survive as parasites to proliferate within human or animal tissues leading to two major life-threatening infectious diseases known as granulomatous amoebic encephalitis (GAE) and *Acanthamoeba* keratitis (AK) that often arise as mild headaches [1,2]. Microscopic-based detection methods are widely used in many countries to detect *Acanthamoeba*; however, these assays are labour intensive, time-consuming, and require *Acanthamoeba* culturing for up to several days [2,3,4]. Recent advances in the *Acanthamoeba* detection technique employs polymerase chain reaction (PCR) assay for the sensitive and rapid detection of *Acanthamoeba* parasites at the genotypic-level [5,6]. PCR technique involves three major steps: denaturation, annealing, and extension to amplify DNA. The DNA polymerase is the crucial ingredient in PCR to synthesize new DNA strands. Despite 90% sensitivity and 90.8% specificity of PCR assay in *Acanthamoeba* detection [3,4,5,6], the challenges of PCR technique include insufficient target DNA yield and non-specific DNA amplification leading to possible band smearing on gels [7]. Previously, *Acanthamoeba* detection used hot-start DNA polymerase enzymes to modify the conventional PCR techniques, avoiding non-specific amplification, and increasing the target DNA yield by inactivating the enzyme at lower temperatures [8]. However, these findings reported 82% specificity, suggesting a possible presence of undesired PCR products as false positives [8]. Additional PCR enhancers used to modify conventional PCR and increase target DNA yield include betaine to amplify GC rich long DNA and dimethyl sulfoxide (DMSO) to improve PCR specificity. However, 2% DMSO addition requires more enzyme because it can inhibit Taq DNA polymerase enzyme in the PCR reaction, affecting the fidelity of the PCR enzyme [9]. Hence, optimizing a PCR technique along with its parameters, is currently a time-consuming technique to obtain an enhanced target DNA yield.

Nanotechnology has emerged with the nanomaterial-assisted PCR (NanoPCR) technique, which introduces nanomaterials into the PCR mixture tto enhance PCR thermal conductivity. Since heat transfer limitation exists in the conventional PCR thermal cycler and is known to reduce PCR reaction efficiency, nano-PCR can address this issue by combining nanomaterials with excellent thermal conductivity properties into a PCR reaction to selectively increase target DNA yield and enhance PCR efficiency [10]. Nanoparticles ranging from 1 to 100 nm in size provide a conducive interface for DNA interactions due to the high surface area to volume ratio. Since nanoparticles possess unique physicochemical properties, significantly higher thermal conductivity, they are efficient PCR enhancers [11]. Graphene nanoparticle was proven to not only enhance PCR specificity but also maintain its fidelity [12]. hBN is an analogue of graphene and exists as a white crystalline solid compound within an ordered arrangement of Boron and Nitrogen atoms in a two-dimensional plane, giving rise to its most stable hexagonal honeycomb layered structure. Hence, the property of hexagonal boron nitride (hBN) is of interest as a potential nanomaterial because of characteristic resemblance to its analogue called graphene. hBN compound is inert and does not oxidize in air up to 900 °C. It possesses high thermal conductivity and is non-toxic in the environment [13], making it highly suitable as a nanoparticle to enhance PCR efficiency.

### Nanoparticle Enhanced Thermal Conductivity

Heat transfer through any liquid depends on its thermal conductivity. PCR reagents, which are water-like substances, have a thermal conductivity of approximately 0.6 Wm^−1^K^−1^. Rate of heating–cooling in PCR tubes is therefore limited by this property of PCR reagents. From an engineering perspective, the only way to address this problem is by introducing substances that have higher thermal conductivities into the PCR reagents. The idea of combining solid-liquid thermal conductivities for higher heat transfer has evolved from the time of Maxwell [14] (Equation (1)) and continues to improve until today.

(1) $$K_{eff}=\frac{k_{p}+2k_{b}+2(k_{p}-k_{b})\emptyset }{k_{p}+2k_{b}-2(k_{p}-k_{b})\emptyset }k_{b}\;\;$$

where *k_eff_* is effective thermal conductivity, *k_p_*, *k_b_*, and *ɸ* are the thermal conductivity of particle, the thermal conductivity of the base fluid, and the volume fraction, respectively. Early efforts failed as the methods were limited to producing micro-sized particles of metals and their densities were higher than the base fluids, causing immediate sedimentation. However, towards the end of the 20th century, nanomaterials were successfully dispersed in to liquids, and that resulted in an anomalous increase in the thermal conductivity. The increase in thermal conductivity is attributed to the Brownian motion of particles, molecular level layering at the liquid-particle interface, nanoparticle clustering [15], the formation of percolation network, and so on [16]. Though Maxwell only took into account the particle volume fraction and conductivities of the particle-fluid, hundreds of analytical and empirical models (for example, Equation (2)) later showed [17,18] how multiple factors influence effective thermal conductivity [19].

(2) $$k_{n}=f\left(\emptyset ,k_{f},\;k_{p},\;v_{Br},\;cp_{f},d_{ref},d_{p},T,v_{f},T_{b}\right)$$

where $k_{n}$, $k_{f}$, $k_{p}$ are thermal conductivity of nanofluid, base fluid, and particle, respectively. $v_{Br}$ is Brownian velocity, ms^−1^; $cp_{f}$ is specific heat of the base fluid, Jkg^−1^K^−1^; $d_{ref}$ is reference diameter; $d_{p}$ is diameter of the nanoparticle, m; T is temperature, K; $v_{f}$ is kinematic viscosity, m^2^s^−1^; and $T_{b}$ is boiling point temperature of the base fluid, K. However, almost all reports on nanoPCR have ignored the effect of nanoparticle thermal conductivity while explaining the observed PCR enhancement. Several metal oxides and carbon allotropes have been explored as PCR enhancers, but hexagonal boron nitride (hBN) is yet to be studied. hBN is a stable, non-metallic dielectric ceramic material exhibiting high thermal conductivity and chemically inert, often referred to as white graphene [20]. It can be produced using different synthesis routes [21] and is relatively cheaper than its 2D counterparts. Similar to many existing reports on various nanofluids, the thermal conductivity of base fluid increases with the increase in the concentration of hBN nanoparticles [22]. It has been shown that 0.1 wt % hBN added to metal stamping lubricant yields over 30% improvement in thermal conductivity [23]. hBN also shows temperature-dependent thermal conductivity, which may be due to the smaller-sized particles undergoing Brownian motion enabling higher energy transport between particles [24]. Therefore, in this research, the objective is to incorporate hBN nanoparticles of an optimum concentration into PCR assays owing to their relatively high thermal conductivity, chemically stable properties, and low cost. Since it can enhance *Acanthamoeba* DNA yield in PCR and improve the efficiency of PCR parameters (e.g., temperature, step, and cycle), it may aid in the rapid detection of *Acanthamoeba*.

## 2. Materials and Methods

### 2.1. hBN Nanoparticle Characterization

The Raman spectra were studied using the Horiba LabRAM HR Evolution Raman spectrometer (Horiba, Kyoto, Japan). The high angle powder X-ray diffraction patterns (XRD) of the hBN were recorded on a Bruker D8 Discover diffractometer with primary monochromatic high-intensity CuK_α_ radiation (λ = 0.15406 nm). The surface structure and elemental composition of the materials were characterized using a field emission scanning electron microscope (FESEM), Hitachi SU8010 (Hitachi, Tokyo, Japan).

### 2.2. Acanthamoeba castellanii Cell Cultures

This study used *Acanthamoeba castellanii* clinical isolate of T4 genotype and was purchased from American Type Culture Collection (ATCC 50492). It was cultured without shaking in 10 mL of proteose peptone yeast extract glucose (PYG) growth medium (proteose peptone 0.75% (w/v), yeast extract 0.75% (w/v), and glucose 1.5% (w/v) in a T-75 tissue culture flask at 30 °C incubator [25]. The growth media was replaced 17–20 h before the ensuing experiment that obtained 95% greater *A. castellanii* trophozoites [26].

### 2.3. Acanthamoeba DNA Extraction Using Chelex, Proteinase K, and Genet Bio Kit Methods

*A. castellanii* cell pellet was maintained in the trophozoite stage in tissue culture flasks in proteose peptone yeast extract glucose (PYG) growth medium. Upon confluene, the flask was chilled on ice for 20 min and unbound *Acanthamoeba* was centrifuged at 2500× *g* for 10 min. The cell pellet was resuspended in 1 mL of fresh proteose peptone yeast extract glucose (PYG) growth medium, and *Acanthamoeba* cell counting was determined by using a hemocytometer in volumes containing 1000, 5000, 10,000, 20,000, and 100,000 cells placed in 1.5 mL Eppendorf tubes. To the above cell number counts, two different methods of DNA extraction were performed, which are 50 µL of chelex (Insta-gene matrix, Bio-Rad, Hercules, CA, USA) and 50 µL of 20 mg/mL of proteinase K enzyme (GeNet Bio, Daejeon, South Korea). In both Chelex and Proteinase K DNA extraction methods, the tubes were incubated at 56 °C, 95 °C, 56 °C, and 100 °C for 45, 10, 60, and 10 min, respectively. Finally, the reaction mixtures of both Chelex and Proteinase K enzyme DNA extraction methods were centrifuged at 2000× *g* for 5 min, and the supernatant containing DNA was used as a template in conventional PCR and then analysed for the presence of *A. castellanii*. DNA band, as described previously [27]. The third DNA extraction method was also performed to the above-stated cell number counts, using the GeNet Bio Kit method based on the manufacturer’s protocol [28]. For Chelex, Proteinase K, and GeNet Bio Kit DNA extraction methods, nano dropping of the supernatant containing the A. castellanii DNA was measured for DNA concentrations. The Chelex method with A. castellanii cell counts of 1000, 5000, 10,000, 20,000, and 100,000 showed DNA concentrations of 22.51, 110.51, 11.67, 40.82, and 151.9 µg/mL, respectively. Their respective DNA purity values were 2.14, 1.72, 4.37, 2.06, and 1.85 A260/280. Next, DNA was diluted to a final volume of 20–50 µL to obtain the DNA concentration of 10 ng/µL. Proteinase K method extracted DNA was not diluted because nano dropping showed no DNA concentrations present. Phosphate-buffered saline (PBS) was added to make it to a final volume of 500 µL as described previously [27]. GeNet Bio Kit method for the above stated cell count numbers obtained less DNA volume compared to Chelex and proteinase K methods with DNA concentrations of approximately 13.77–14.21 µg/mL with DNA purity value of approximately 2.80 A260/280.

### 2.4. Preparation of hBN Nanofluid

hBN nanoparticles of ∼70 nm were dispersed in autoclaved double distilled water to prepare a solution of 10 mM final concentration. For proper mixing of hBN nanoparticles in water, sonication was performed for six hours using a bath-sonicator equipment (50–60 Hz, 50 W; 230 V; ultrasonic bath XUBA1 Grant Ltd.). This well dispersed hBN nanofluid was used as a stock solution to appropriately dilute a range of various hBN concentrations (0.4–1 × 10^−^^8^ wt % hBN) similar to our previous report [29]. The thermal conductivity of the samples was measured using a KD2 pro conductivity meter identical to the methodology used in our earlier studies [30].

### 2.5. PCR, Nano-PCR, and Gel Electrophoresis

For *Acanthamoeba*, PCR was performed in 25 μL of the total volume containing 1 μL of 1 Unit Prime DNA Taq polymerase (Genet Bio), 1 μL of 10 ng/µL. DNA, 1 μL of 2 mM dNTPs mixture, 8.5 μL of nuclease-free water, 1 μL of 25 μM MgCl_2,_ and 1 μL of each 10 μM forward and reverse *Acanthamoeba* genus-specific primer sequences (Table 1) [31]. PCR thermocycler reaction involved initial denaturation at 94 °C for 3 min followed by 40 cycles of denaturation at 94 °C for 30 s, annealing at 50 °C for 30 s, extension at 72 °C for 30 s, and a final extension at 72 °C for 10 min with an expected PCR amplified product size of 950 bp for *A. castellanii* [28]. PCR assay was repeated 3 times to observe the consistency of visible band strength.

For nano-PCR, hBN nanoparticles were added into PCR reactions to enhance the amplification of T4 genotype DNA using various hBN concentrations (0.4–1 × 10^−^^8^ wt % hBN). To determine optimum hBN concentration, nano-PCR involved PCR protocol parameters, as described previously [28]. Next, to improve PCR efficiency in the rapid detection of *Acanthamoeba*, nano-PCR used hBN optimum concentration to test the effects of PCR parameters (e.g., temperature, step, and cycle). Hence, we reduced temperatures in denaturation, annealing, and extension over a range of 5 °C each, while keeping the step and cycle time constant as previous [28]. Furthermore, using the same optimum hBN concentration, we reduced the nano-PCR step time of denaturation, annealing, and extension by five seconds each, while keeping temperature and cycle constant as previous [28]. Next, we reduced the number of cycles up to 29 times from previously stated 40 times to reduce time in *Acanthamoeba* detection. After PCR and nano-PCR, 5 µL of equal PCR products were electrophoresed along with a reference DNA ladder of 1 kB (GeneDireX^®^, Bio-Helix Co, Ltd.) on 1% agarose gels with 1× TAE buffer and stained with ethidium bromide. Gel electrophoresis of PCR products ran at 100 V for 45 min. PCR products were visualized under UV illumination [32].

### 2.6. Quantitative Analysis of Amplified Nano-PCR Products

The amounts of amplified PCR products were quantified using NIH Image J software to measure the band density by scanning the gel pictures generated. The band intensities were expressed as arbitrary units (a.u.). Error bars were included using the function “standard error” in the Excel spreadsheet.

## 3. Results

### 3.1. Nanoparticle Characterization

**Figure 1:**
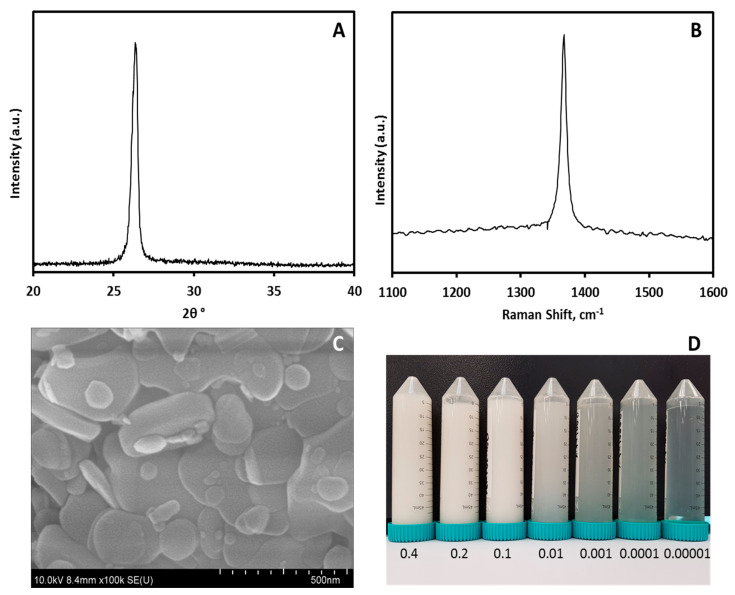
(**A**) Powder X-ray diffraction (XRD) on hBN nanoparticles at atmospheric pressure. (**B**) Raman Spectra of hBN nanoparticles. (**C**) Field Emission Scanning Electron Microscopic (FESEM) image of hBN measured at 10 kV vacuum condition. (**D**) Serially diluted samples of hBN-ddH_2_O from a stock of 0.4 wt %.

Figure 1A shows the XRD pattern of the hBN nanoparticle used in this work. The peak originating from hBN were observed at 2θ angles of 26.8°, and the nanoparticles display high crystallinity owing to its narrow peak. The observed peak can be indexed to (002) of hBN Joint Committee on Powder Diffraction Standard (JCPDS) No.34-0421. The sample was further confirmed using Raman spectra (Figure 1B), which shows a sharp peak at 1363 cm^−^^1^. This indicates good quality of hBN used in this study involving the vibration mode of its intralayer E_2g_. The FESEM image of hBN shown in Figure 1C shows heterogeneity of its flake size. The flakes are distributed within the range of approximately 50 nm to 1 micron. The flakes do not look agglomerated and could be easily dispersed in base fluid with the help of sonication.

### 3.2. Thermal Conductivity

**Figure 2:**
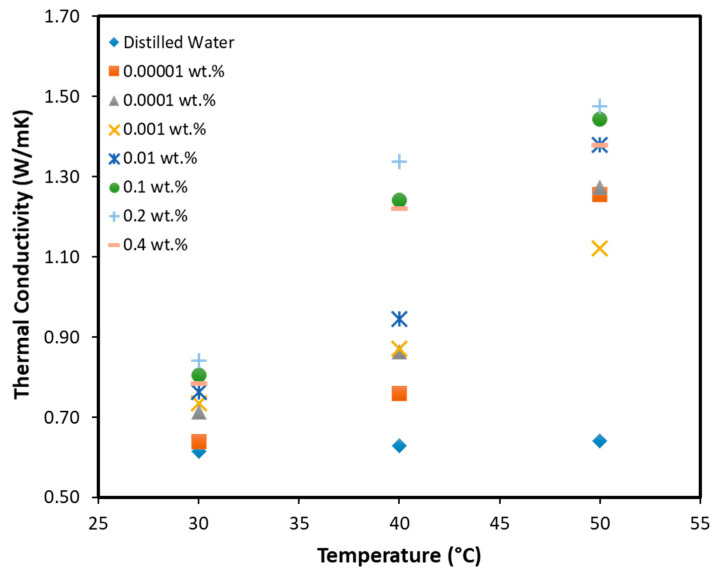
Thermal conductivity measured at three different temperatures of dd-H2O and a range of hBN-ddH2O concentration.

Convective heat transfer is a function of the thermal conductivity of the fluid. The thermal conductivity measurements on nanofluids have shown that the conductivity is inversely proportional to the size of the nanoparticle. Other possible reasons for enhanced thermal conductivity include Brownian motion of particles, molecular level layering at the liquid-particle interface, the formation of percolation network, and nanoparticle clustering [15]. Figure 2 shows the thermal conductivity of hBN nanofluids with respect to increasing temperature at all the concentrations used in this study. Results indicate that the thermal conductivity of the samples is a vital function of particle concentration and temperature. The highest enhancement in thermal conductivity was 141.9 W/mk with 0.2 wt % hBN. High thermal conductivity could also be attributed to the highly crystalline nature of the hBN powder used in this study, which might induce longer phonon scattering mean free path [33]. Further increase in the concentration may not help since agglomeration would result in sedimentation affecting the thermal conductivity unless the particles are functionalized [34]. It is also proposed that the higher concentration leads to nanoparticle populated network, which is not as efficient as the co-existence of dynamic free particles and local clusters, which could lead to lower conductivity at higher temperatures. Moreover, the higher concentration would also contribute to the increase in heat capacity of the reaction, which may affect the yield and reaction fidelity.

### 3.3. Chelex Assay with 10,000 Acanthamoeba Cells Was Optimized among Three DNA Extraction Methods Tested to Identify Low Cell Numbers

**Figure 3:**
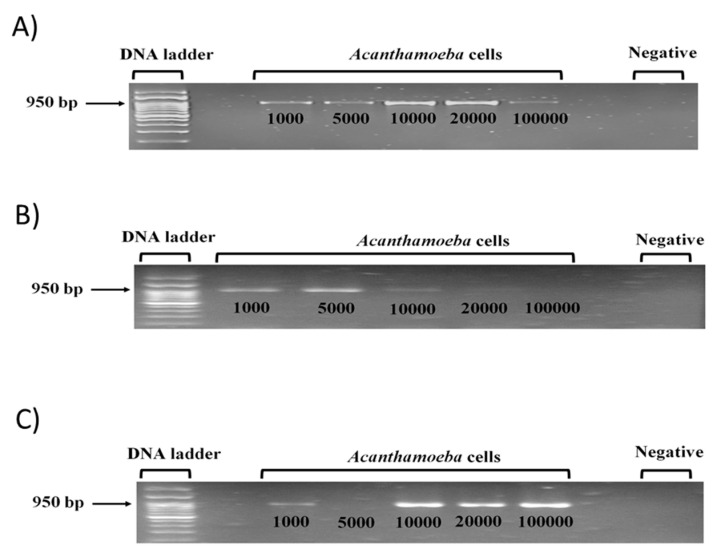
Agarose gel image showing PCR amplification of 18S rDNA of *A. castellanii* of the T4 genotype using three different DNA extraction methods, (**A**) Chelex assay, (**B**) Proteinase K assay, and (**C**) GeNet Bio Kit assay.

*Acanthamoeba* T4 genotype DNA were observed for PCR bands in various cell numbers of 1000, 5000, 10,000, 20,000, and 100,000, which obtained the expected 950 bp amplicon (Figure 3A–C). For clinical purposes, a sensitive detection assay is essential to identify *Acanthamoeba* at low cell numbers. Chelex assay consistently obtained sharp, bright PCR bands under 20,000 cells as no DNA loss occurs via washing steps (Figure 3A). Proteinase K assay showed PCR bands in >5000 cells with low DNA yield implicating low fidelity of this enzyme due to heat inactivated degradation (Figure 3B). GeNet Bio Kit assay showed bright PCR bands for >10,000 cells, but PCR band was absent at lower 5000 cells indicating loss of DNA caught on the filter during the washing process (Figure 3C). No band was present for the negative control, hence no contamination occurred. Note chelex assay with 10,000 *A. castellanii* cells in 10 ng/µL. concentration was selected as optimum (Figure 3A).

### 3.4. HBN Nanoparticle Enhances Acanthamoeba Detection in PCR and Increases PCR DNA Yield

**Figure 4:**
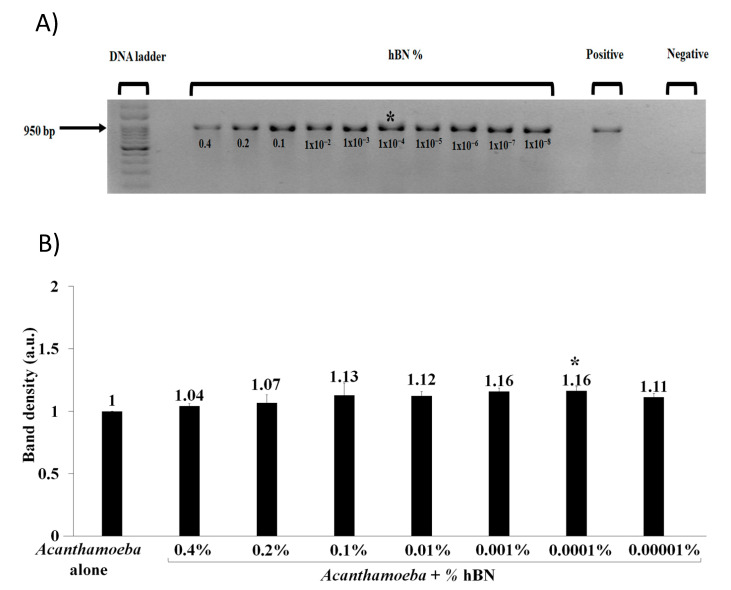
(**A**) Agarose gel image showing concentration-dependent hBN nanoparticles effects on PCR amplification of *A. castellanii*. Lane “hBN %” indicates 0.4–1 × 10^−8^ wt % hBN nanoparticle concentration in PCR along with 10,000 *A. castellanii* cells enhanced PCR products in gradual increments of DNA yield. An expected amplicon size of 950 bp was obtained. Lane positive control indicates PCR reaction with a DNA template but without hBN nanoparticles. Lane negative control indicates PCR reaction with distilled water but without added DNA template and hBN nanoparticles. (**B**) Quantitative graph of 950 bp *Acanthamoeba* PCR band density in the presence of (0.4–1 × 10^−8^ wt %) hBN nanoparticles against the absence of hBN nanoparticles. The PCR amplicon in the absence of nanoparticles was considered as 1 arbitrary unit (a.u.) compared to amplicons in the presence of hBN nanoparticles as relative amounts of PCR yield increase. Note hBN nanoparticles improved *Acanthamoeba* PCR yield by ~16% at maximum optimum concentration of 1 × 10^−4^ wt % indicated in both (**A**) and (**B**) with (*).

hBN nanoparticles with a concentration of 0.4–1 × 10^−^^8^ wt % were added to the PCR reaction mixture containing 10,000 *A. castellanii* cells to test the effects of hBN on *Acanthamoeba* detection. Gel image results revealed that 1 × 10^−^^4^ wt % hBN optimum nanoparticle concentration enhanced *Acanthamoeba* PCR DNA yield (Figure 4A). ImageJ software showed that 1 × 10^−^^4^ wt % hBN optimum nanoparticle concentration resulted in a 1.16-fold increase in band density (Figure 4B). Out of the eight trials carried out to establish the optimal concentration, most of the bands indicated to 1 × 10^−4^ wt % hBN as the optimal concentration. However, possibly due to human error while loading PCR product in the agarose gel, a perfect bell pattern could not be achieved in the graph based on image intensity quantification. The best gel image is presented in (Figure 4A) as a representation of the effect of nanoparticle concentration.

### 3.5. hBN Nanoparticle Reduces PCR Temperature to Enhance Acanthamoeba Detection and Increases DNA Yield

**Figure 5:**
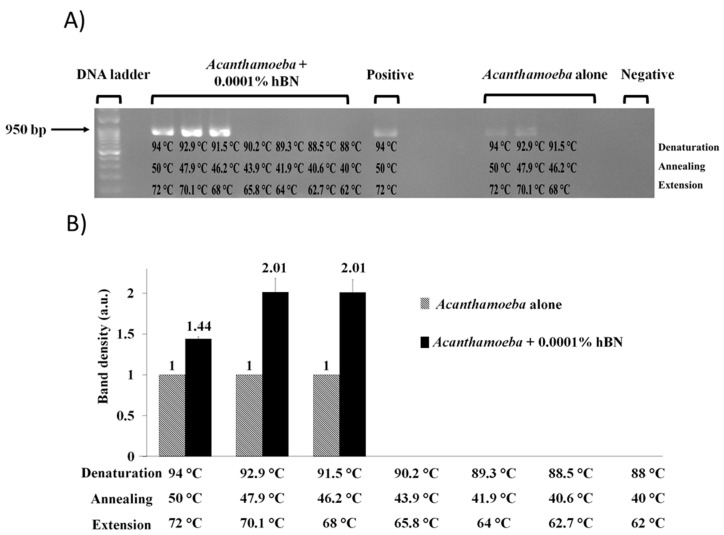
(**A**) Agarose gel image showing a range of reduced temperature-dependent effects on PCR amplification of *A. castellanii*. Lane “*Acanthamoeba* + 0.0001% hBN” indicates 1 × 10^−4^ wt % optimum hBN concentration along with 10,000 *A. castellanii* enhanced *Acanthamoeba* PCR products in gradual increments of DNA yield at reduced PCR temperatures for denaturation, annealing, and extension. An expected amplicon size of 950 bp was obtained. Lane positive control and “*Acanthamoeba* alone” indicates PCR reaction with DNA template but without hBN nanoparticle. Lane negative control indicates PCR reaction with distilled water but without added DNA template and hBN nanoparticle. (**B**) Quantitative graph of 950 bp *Acanthamoeba* PCR band density in the presence of hBN nanoparticle against the absence of hBN nanoparticles at reduced PCR temperatures for denaturation, annealing and extension. The PCR amplicon in the absence of nanoparticles was measured as 1 arbitrary unit (a.u.) compared to amplicons in presence of hBN nanoparticles as relative amounts of PCR yield increase. Note 1 × 10^−4^ wt % optimum hBN nanoparticle concentration improved *Acanthamoeba* PCR yield by ~100% at lowest 46.2 °C annealing temperature.

To test the effects of hBN nanoparticle on *Acanthamoeba* detection at reduced PCR temperatures for denaturation, annealing, and extension an optimum nanoparticle concentration was added to PCR reaction mixture containing 10,000 *A. castellanii* cells. Gel image results revealed 1 × 10^−^^4^ wt % hBN optimum nanoparticle concentration enhanced *Acanthamoeba* PCR DNA yield at 46.2 °C low annealing temperature as primers effectively hybridized to target gene; followed by low denaturation and extension temperatures at 91.5 °C and 68 °C, respectively. However, at this same annealing temperature, no DNA amplification occurred for *Acanthamoeba* positive control without hBN nanoparticle (Figure 5A). Quantification of band density in gel images showed that 1 × 10^−^^4^ wt % hBN optimum nanoparticle concentration resulted in ~2.0-fold increase in band density at 46.2 °C low annealing temperature (Figure 5B).

### 3.6. hBN Nanoparticle Reduces PCR Step Time to Enhance Acanthamoeba Detection

**Figure 6:**
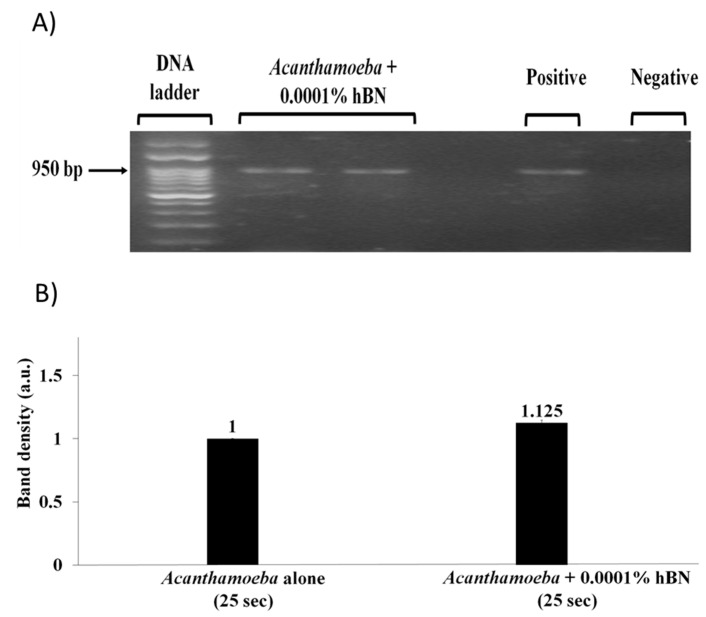
(**A**) Agarose gel image showing effects of reduced PCR step time on PCR amplification of *A. castellanii*. Lane “*Acanthamoeba* + 0.0001% hBN” indicates 1 × 10^−4^ wt % optimum hBN concentration along with 10,000 *A. castellanii* cells did not cause any decrease in the amount of amplified *Acanthamoeba* PCR products at reduced PCR step time from 30 to 25 s for denaturation, annealing, and extension. An expected amplicon size of 950 bp was obtained. Lane “*Acanthamoeba* alone” indicates PCR reaction with DNA template but without hBN nanoparticle. Lane negative control indicates PCR reaction with distilled water but without added DNA template and hBN nanoparticle. (**B**) Quantitative graph of 950 bp *Acanthamoeba* PCR band density in the presence of hBN nanoparticles against the absence of hBN nanoparticles at reduced PCR step time for denaturation, annealing, and extension. The PCR amplicon in the absence of nanoparticles was measured as 1 arbitrary unit (a.u.) compared to amplicons in the presence of hBN nanoparticles as relative amounts of PCR yield increase. An optimum hBN nanoparticle concentration of 1 × 10^−4^ wt % partially improved *Acanthamoeba* DNA yield at 25 s.

To test the effects of hBN nanoparticle on *Acanthamoeba* detection at reduced PCR step time for denaturation, annealing, and extension an optimum nanoparticle concentration was added to PCR reaction mixture containing 10,000 *A. castellanii* cells. Gel image results revealed that reduction in PCR step time from 30 to 25 s using 1 × 10^−^^4^ wt % hBN optimum nanoparticle concentration did not cause any decrease in the amount of amplified PCR product compared to the *Acanthamoeba* positive control without hBN nanoparticle (Figure 6A). Quantification of band density in gel images showed that 1 × 10^−^^4^ wt % hBN optimum nanoparticle concentration resulted in partial improvement of *Acanthamoeba* DNA yield at 25 s (Figure 6B).

### 3.7. hBN Nanoparticle Reduces PCR Cycle Time to Enhance Acanthamoeba Detection

**Figure 7:**
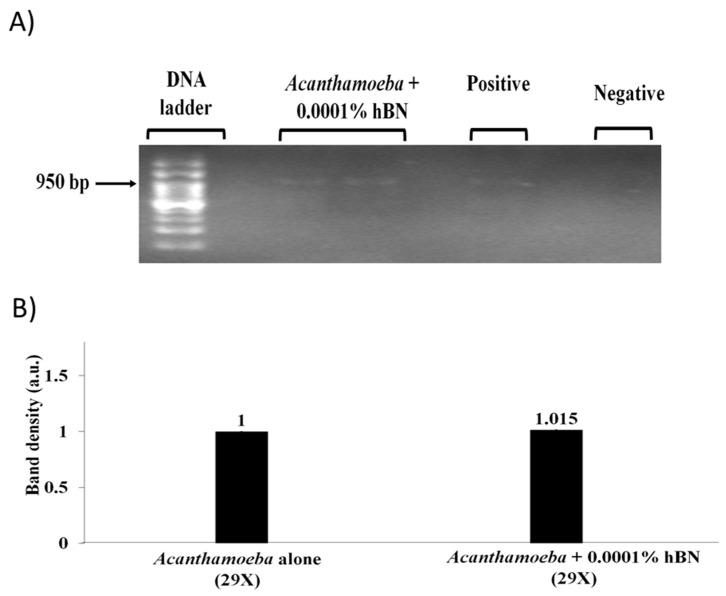
(**A**) Agarose gel image showing effects of reduced PCR cycle time on PCR amplification of *A. castellanii*. Lane “*Acanthamoeba* + 0.0001% hBN” indicates 1 × 10^−4^ wt % optimum hBN concentration along with 10,000 *A. castellanii* cells did not cause any decrease in the amount of amplified *Acanthamoeba* PCR products at reduced PCR cycle number from 40× to 29× for denaturation, annealing, and extension. An expected amplicon size of 950 bp was obtained. Lane “*Acanthamoeba* alone” indicates PCR reaction with DNA template but without hBN nanoparticle. Lane negative control indicates PCR reaction with distilled water but without added DNA template and hBN nanoparticle. (**B**) Quantitative graph of 950 bp *Acanthamoeba* PCR band density in the presence of hBN nanoparticles against the absence of hBN nanoparticles at reduced PCR cycles from 40× to 29×. The PCR amplicon in the absence of nanoparticles was measured as 1 arbitrary unit (a.u.) compared to amplicons in presence of hBN nanoparticles as relative amounts of PCR yield increase. An optimum hBN nanoparticle concentration of 1 × 10^−4^ wt % partially improved *Acanthamoeba* DNA yield at 29×.

To test the effects of hBN nanoparticle on *Acanthamoeba* detection at reduced PCR cycle time an optimum nanoparticle concentration was added to PCR reaction mixture containing 10,000 *A. castellanii* cells. Gel image results revealed that reduction in PCR cycle number from 40× to 29× using 1 × 10^−^^4^ wt % hBN optimum nanoparticle concentration did not cause any decrease in the amount of amplified PCR product compared to the *Acanthamoeba* positive control without hBN nanoparticles, suggesting that hBN nanoparticles can rapidly reduce overall PCR cycle number by 11× and time by 32 min; thus, enhancing *Acanthamoeba* detection (Figure 7A). Quantification of band density in gel images showed that 1 × 10^−^^4^ wt % hBN optimum nanoparticle concentration partially improved *Acanthamoeba* DNA yield at 29 cycles (Figure 7B).

## 4. Discussion

In most clinical cases, detection of the *Acanthamoeba* T4 genotype prevalent in 94% *Acanthamoeba* keratitis eye infection cases is delayed because bacterial, viral, and fungal keratitis are primarily tested [6]. For example, lethal ring infiltrate developed in *Acanthamoeba* keratitis patient due to treatment initiated for bacterial keratitis. Hence, a highly sensitive detection method is required to isolate *Acanthamoeba* from low amoeba cell density to prevent exacerbation of disease symptoms. Currently, PCR assay is used in *Acanthamoeba* detection. However, insufficient DNA yield [1] and reduced PCR specificity in Acanthamoeba DNA amplification suggests that non-specific amplification remains an issue [8]. Note, chelex assay with 10,000 A. castellanii cells in 10 ng/µL concentration was selected as the optimum cell count number to react with hBN in the PCR reaction mixture. GeNet Bio Kit assay showed the presence of bright PCR bands for samples; however, the PCR band was absent at lower 5000 cells indicating loss of DNA, which was perhaps caught on the filter during the washing process. Chelex assay consistently obtained sharp, bright PCR bands for all the samples, which means no DNA loss occurred because there were no washing steps involved like the GeNet Bio Kit assay. Chelex involves fewer steps to extract the DNA, which makes it less prone to errors in losing the DNA compared to GeNet Bio Kit method.

In particular, nanoparticles have been developed as a PCR enhancer to improve PCR efficiency. Many nanoparticles have been developed to enhance PCR specificity and improve PCR DNA yield, such as TiO_2_, gold, and graphene [10]. As hBN is firstly cheaper, present in higher abundance, inert (does not oxidize in air up to 900 °C) and is highly resistant to chemical attacks, it exhibits weak interaction with other chemical substances (i.e., metabolites or phenolic compounds) present in amoeba samples from clinics and environment; thus, it is a nanoparticle of choice to improve *Acanthamoeba* PCR amplification. Interestingly, in our study, 70 nm hBN nanoparticles of as low optimum concentration of 1 × 10^−^^4^ wt % showed enhanced PCR yield by ~16% for 10,000 *Acanthamoeba* cells (Figure 4B). This observation is comparable to the enhanced DNA yield of *Acanthamoeba* in PCR assay using graphene, copper, and alumina nanoparticles, as reported in our previous study [28]. Given the high surface area to volume ratio of 70 nm hBN nanoparticles, it provides greater surface availability for adsorption and desorption of small primers, DNA template, and DNA polymerase enzyme with enhanced reactivity nanomaterial surface [7]. Thus, the high surface area property of hBN nanoparticles is advantageous to generate a greater number of PCR amplicons with a low amount of (10 ng/µL.) template concentration compared to amoeba positive control without nanoparticle (Figure 4A) that improves PCR efficiency in *Acanthamoeba* detection.

Nanoparticles have high thermal conductivity to enhance PCR specificity and DNA yield [7]. hBN nanoparticle is thermally stable with high thermal conductivity theoretical value of 600–1000 W m^−1^ K^−1^ [13]. Interestingly, in our study, hBN nanoparticle of 1 × 10^−4^ wt % optimum concentration resulted in a ~2.0-fold increase of *Acanthamoeba* PCR yield at a low annealing temperature of 46.2 °C (Figure 5B). This observation is consistent with graphene nanoparticles in the PCR assay, which enhanced human mitochondrial DNA yield at a low annealing temperature of 25 °C [12]. The low annealing temperature is vital to reduce non-specific amplification and increase PCR specificity because primers must effectively hybridize to target genes and not with any other similar sequences [9]. However, in conventional PCR, a very low annealing temperature is not favourable because non-uniform heat distribution occurs among PCR reagents due to repeated heating and cooling process of PCR thermocycler that may cause non-specific amplification and low DNA yield. Hence, we have observed that hBN nanoparticle showed a bright PCR band at low annealing temperature of 46.2 °C due to enhanced heat transfer as well as uniform heat distribution to *Acanthamoeba* primers and DNA template; whereas, no DNA amplification was shown at similar annealing temperature for *Acanthamoeba* positive control without hBN nanoparticle (Figure 5A). Thus, the heat transfer property of hBN nanoparticle is advantageous to generate a greater number of amplicons and improve PCR efficiency in *Acanthamoeba* detection. The presence of nanoparticle around the PCR reagents would augment Brownian motion induced heat transfer. In case of agglomerated particles distributed and in contact with individual 2D flakes, they would also create a path for rapid heat transfer.

Reduction of PCR step time from 30 to 25 s in denaturation, annealing, and extension observed Acanthamoeba PCR bands using 1 × 10^−4^ wt % hBN optimum nanoparticle concentration. There was no observed decrease in the amount of amplified PCR product with hBN compared to Acanthamoeba positive control without hBN nanoparticle, suggesting that overall PCR step time can be reduced by 10 min; thus, enhancing Acanthamoeba detection (Figure 6B). Similarly, 11 cycles were reduced from initial 40× cycles from Figure 4B using 1 × 10^−4^ wt % hBN optimum concentration to 29× using 1 × 10^−4^ wt % hBN optimum nanoparticle concentration which saves 32 min in Acanthamoeba detection time, without any decrease in amplified PCR product compared to positive control (Figure 7B). The clinical diagnosis maybe expedited with a reduction in PCR reaction time. The partial DNA enhancement in both PCR step and cycle reaction may attribute to reduced number of PCR reactions occurring per cycle. Nevertheless, PCR bands were still observed with hBN at a much shorter time indicating that hBN nanoparticles did not inhibit PCR reaction for low amounts of Acanthamoeba 10 ng/µL. template concentration. Interestingly, bare hBN nanoparticles were reported to be non-cytotoxic to osteoblast bone cells and macrophage cells evaluated for hBN suitability in orthopedic implants [35], suggesting that hBN is likely non-toxic to DNA.

**Figure 8:**
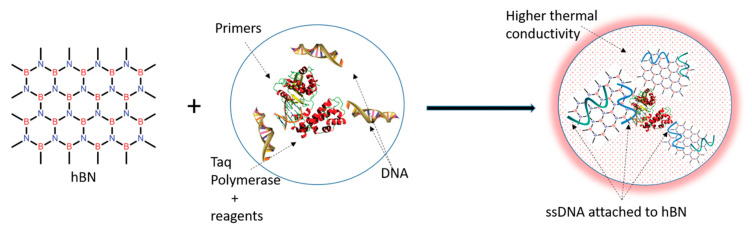
Graphical representation hBN induced enhanced thermal conductivity of PCR.

Although the precise mechanism of action between hBN nanoparticle and PCR reagents requires further study, enhancement of *Acanthamoeba* PCR DNA yield is possibly due to high adsorption affinity of hBN nanoparticles to purine (Guanine—G) [36], which is favorable to break strong GC rich hydrogen bonds of double stranded DNA template in PCR denaturation step. It was also reported that DNA nucleobases retained stable configurations when hBN nanoparticle surface adsorbed the DNA template [37]. Polar hBN nanoparticles adsorb negatively charged phosphate backbone of primers and DNA templates in the PCR annealing step due to electrostatic interactions, as depicted in the graphical illustration, Figure 8. Desorption of attached nucleic acids occurs when complementary sequences are present, which prevents non-specific amplification, resulting in enhanced PCR specificity. Finally, DNA amplification occurs in the PCR extension step due to a more significant catalytic activity of DNA polymerase enzyme [7]. The entire process of adsorption and desorption of DNA strands over nanoparticles must have also been strongly influenced by the augmented heating–cooling rates due to hBN. The higher thermal conductivity achieved in the PCR mixture due to the addition of hBN could have been a result of Brownian motion of hBN, liquid layering around the flakes, percolation network of hBN flakes and its agglomerates, and thermophoretic effect. Nonetheless, further studies on each mechanism highlighted here are required to establish a factual basis for nanoparticle enhanced PCR reactions.

In conclusion, hexagonal boron nitride (hBN) nanotechnology-based PCR improves PCR DNA yield in *Acanthamoeba* detection and can be useful for clinical applications or water companies in rapid detection of *Acanthamoeba.* An optimal concentration of hBN, 0.4–1 × 10^−8^ wt % increases the DNA yield. The fidelity of PCR with hBN is a function of its concentration. The addition of hBN significantly helps in reducing the reaction step time, number of cycles, and temperature without compromising the yield with respect to positive control. Enhanced thermal conductivity induced adsorption and subsequent desorption of DNA over hBN is proposed to be the underlying mechanism of the enhancements observed in this study. However, it requires further investigation, particularly from the better PCR yield quantification point of view.

## Figures and Tables

**Table 1:** Oligonucleotide primers used for *Acanthamoeba* PCR amplification.

Protist	Gene-Specific Primers	Primer Properties	Amplicon	Reference
*Acanthamoeba*	Forward primer 5′-TTTGAATTCGCTCCA ATAGCGTATATTAA-3′ Reverse primer 5′-TTTGAATTCAGAAA GAGCTATCAATCTGT-3′	Tm: 55.1 °C GC content: 31% Tm: 55.1 °C GC content: 31%	950 bp	(Kong and Chung, 1996) [31]
